# Comparison of a French pediatric type 1 diabetes cohort’s responders and non-responders to an environmental questionnaire

**DOI:** 10.1186/1471-2458-14-1241

**Published:** 2014-12-03

**Authors:** Sophie Le Fur, The Isis-Diab collaborative group, Pierre Bougnères, Alain-Jacques Valleron

**Affiliations:** Institut National de la Santé et de la Recherche Médicale (U986), Paris Sud University, Le Kremlin Bicêtre, France; Department of Pediatric Endocrinology, Bicêtre Hospital, Le Kremlin Bicêtre, France; Isis-Diab collaborative group, Inserm U986, Le Kremlin Bicêtre, France

**Keywords:** Type 1 diabetes, Children, Questionnaire, Responders, Environment

## Abstract

**Background:**

Type 1 diabetes (T1D) incidence has doubled since the 1980’s for children aged <5 years old, potentially relevant environmental factors having thus to be sought early in the patient’s life. The identification of environmental factors that can explain the changing epidemiology of T1D requires comprehensive environmental inquiries. However, a limitation is the willingness of patients and families to complete these environmental questionnaires. Our objective was to identify patients’ personal and social characteristics predictive of the return, time to the return and completeness of a comprehensive environmental questionnaire.

**Methods:**

The parents of 2832 T1D patients aged <15 years old enrolled in the French Isis cohort were sent a 1379-item environmental questionnaire. A geographic information system was used to collect information on patients’ socioeconomic environment. Multivariate statistical analyses were conducted to identify predictors of questionnaire return, time to its return and its completeness.

**Results:**

Within 6 months, 867 (30.6%) questionnaires were returned. Socioeconomic environment was strongly associated with the probability of response, with fewer responses from cities with high Townsend deprivation index (p =2 × 10^−7^), high unemployment (p =0.005), blue-collar workers’ rate (p =0.0002) and household overcrowding (p =0.02). Response rates were similar for male and female patients, but were higher for less severely affected patients (p =0.006) and younger patients (p =5 × 10^−5^). When returned, completeness was high with a mean of 96%.

**Conclusion:**

Identification of personal or socioeconomic characteristics differing between questionnaire responders and non-responders may help target future environmental investigations on those patients who will more likely return the information, and reduce bias using these variables to stratify the analyses.

**Electronic supplementary material:**

The online version of this article (doi:10.1186/1471-2458-14-1241) contains supplementary material, which is available to authorized users.

## Background

While childhood type 1 diabetes (T1D) was uncommon during the first half of the 20^th^ century, its incidence has risen in Europe over the past 50 years [1], too rapidly to be explained by genetic factors alone. In several European countries, T1D incidence continues to progress rapidly and has doubled since the 1980’s for children aged <5 years old [2]. Thus, potentially relevant environmental factors have to be sought early in the patient’s life. Environmental factors contributing to a multifactorial disease can have a direct and immediate effect on pathological pathways, and can also act at the genomic level through persistent epigenomic changes that can influence the expression of genes relevant to disease pathogenesis. Such epigenomic changes may occur in early prenatal and postnatal life, a period of intense epigenetic activity, or even during the parents’ gametogenesis [3]. When searching for factors possibly associated with T1D, one should thus focus on the environment of the child before overt disease onset, and the mother before and during her pregnancy.

Previous case–control studies of environmental associations with T1D examined specific candidate factor approaches [4–8], but no single factor has been substantiated as causative of T1D [9, 10].

Knowing that environmental factors play an important role in causing T1D, and in light of the paucity of clear results, a comprehensive analysis of the environment screening for all possible causes or combination of causes is sorely lacking. Such an environment-wide association study [11] echoes the systematic, and data-driven search of genetic factors with genome-wide association studies. The most straightforward way to obtain this environmental information is to use questionnaires. A face-to-face approach is hardly feasible when information has to be acquired on hundreds of variables (as done in the example detailed herein). Hence, mailed questionnaires are frequently used to collect the information, but are known to be associated with low percentages of returns, which may represent a bias [12–16]. A large body of literature addresses importance of reducing non-response bias in adult inquiries, but non-response in research on children has been studied less extensively [17, 18]. Conflicting findings were reported concerning the influence of parental (age, culture, ethnicity, language, education level, income, wellbeing and stress) and family factors (environment, structure and familial disease history), and the importance of the nature and severity of the disorder affecting the child [17, 19, 20].

Herein, we describe our inquiry experience with an environmental questionnaire sent to the parents of a large cohort of T1D children, and show that it was possible to identify patients’ personal and socioeconomic characteristics predictive of document return, the time to its return, and its completeness. Analysis of the information provided was then used to maximize the return of information and evaluate the impact of non-response on study outcomes.

## Methods

### Population study

The studied population comprised participants in Isis-Diab cohort. Isis-Diab is an ongoing prospective cohort of French T1D patients recruited since 2007 by the Isis-Diab Network composed of 99 diabetes centers covering almost all French regions (see Additional file 1: Table S1) to investigate environmental factors in the context of the genetic susceptibility to T1D. Environmental causes of childhood T1D are tracked by modeling a wide-scope, « large net » systematic approach aimed at characterizing as many items as possible in the patients’ environment and lifestyle, described as an « environment scan » of the individual environmental variables. Thus, the data collection includes at entry a comprehensive 1374-items environmental questionnaire for all subjects and a full genotyping with Illumina biochips. The present study provides an essential milestone in the environmental analysis of the Isis-Diab project, as it will help define how the questionnaire’s results could be extrapolated to all the T1D patients of the Isis-Diab cohort. Inclusion criteria for the current study were T1D occurring in children <15 years old. T1D was defined according to the American Diabetes Association [21], and by positive autoantibodies to glutamic acid decarboxylase, insulin, and/or islet antigen-2. All studied patients were born in France. Patients were included in the study according to the French bioethics law with families being carefully informed and having signed a detailed informed consent agreed by CPP (number DC-2008-693; NI 2620, Comité de Protection des Personnes). ClinicalTrial.gov identifier: NCT02212522.

### The environmental questionnaire

The questionnaire contained 1379 items about the wider environment (health, nutrition, habitat, social environment and interactions, recreation, animals). Responses to the 562 core questions of the questionnaire were analyzed in this study: 40 questions addressing the period prior to the patient’s mother pregnancy (part 1), 98 questions about the pregnancy (part 2), 61 concerning the delivery and early post-natal life of the T1D patient (part 3), and 363 questions on environmental factors during the patient’s childhood until diabetes was diagnosed (part 4). The other 817 questions were conditioned by the answer to a core question (e.g., if the mother had been exposed to a domestic animal during pregnancy (core question), additional questions concerned the nature of the animal).

Questionnaires were sent to all parents of 2832 T1D children <15 years old enrolled in the Isis-Diab cohort, during the month following their inclusion in the study. Parents were asked to complete the questionnaire at home and send it back in a pre-paid enveloppe. We define responders as all those that returned their questionnaire within 33 months which was the longer follow-up available at the time of this paper, and non-responders as those that did not return their questionnaire within that time. All parents having provided a phone number were contacted once during the week following the questionnaire sending. If there is no return within 3 months, parents receive a reminder by mail.

### Personal data

#### Demography

Sex, age at T1D diagnosis, age and diabetes duration when the questionnaire was sent.

#### Diabetes control

Glycated hemoglobin (HbA1c) and daily insulin dose at the first clinical visit 1–6 years after diabetes diagnosis (these limits were chosen to avoid the honeymoon period during the disease’s first year and the potential heterogeneity introduced by patients enrolled in the cohort long after diabetes onset).

#### Socioeconomic environment

Geolocalization of the patients’ addresses was done using the ArcGIS 9.3.1 system, the ArcView software, and the BD ADRESSE® V2 database provided by the French National Geographic Institute (http://professionnels.ign.fr/bdadresse). Each patient’s socioeconomic environment was estimated by linking their geocoded place of residence at time of their enrolment in the Isis-Diab cohort to anonymous public databases (French Quetelet Network (http://www.reseau-quetelet.cnrs.fr), via the Centre Maurice Halbwachs – Archives de Données Issues de la Statistique Publique (http://www.cmh.greco.ens.fr/adisp.php)). The estimated population density was that of INSEE (2010 census) in the 1 km × 1 km surrounding the patient’s address. 2007 databases were used for the other variables (census closest to the date that patients started to receive the environmental questionnaire). The other variables were defined at the level of the patient’s “commune” (town) of residence, the smallest French administrative entity, LAU2 (Local Administrative Units) according to European Union definition [22]; there are 36,680 communes in continental France (total area: 550,000 km^2^).

The following variables were used to characterize the socioeconomic environment of each patient: urban units index (as a code reflecting the size of the commune’s urban area), unemployment (as a percentage of all individuals ≥16 years old who are economically active); blue-collar workers (as a percentage of all households); white-collar workers (as a percentage of all households); non-car ownership (as a percentage of all households); farmer (as a percentage of all households); household overcrowding (households with >1 person per room, as a percentage of all households); non-home ownership (as a percentage of all households); mean income by year; access rate to high school diploma; the Townsend deprivation index (TDI) was devised in 1988 [23] to assess socioeconomic status. That index is based on 4 variables taken here from the 2007 French INSEE (Institut National de la Statistique et des Etudes Economiques) census: unemployment, non-car ownership, non-home ownership and household overcrowding, that are combined to form an overall score, according to a formula described in [23]. A higher TDI score implies more severe deprivation.

Geographic origin was self-reported for 2390 patients according to the 4 grandparents’ birthplaces. In this report, participants of European descent (4 European grandparents) are used as the reference group in analyses and compared to all others participants (for information, non-European participants were mostly from the North Africa).

### Scores predicting environmental questionnaire return

Two different scores were calculated. The first, a score of the unwillingness to return the environmental questionnaire (henceforth the unwillingness score), was obtained by selecting the variables of Tables 1 and 2 achieving p <0.05 in a global logistic-regression analysis and by assigning each a weight corresponding to deviance residue: [(TDI * 18.6) + (unemployment * 6.2) + (blue-collar workers * 12.8) + (non-homeowner * 5.4) + (geographic origin * 16.9) + (age when questionnaire completed * 14.5) + (HbA1c * 9.3) + (diabetes duration * 10.8))/10]. Minimum and maximum limits were 21 and 144 respectively for this unwillingness score (mean =81.3).

**Figure 1:**
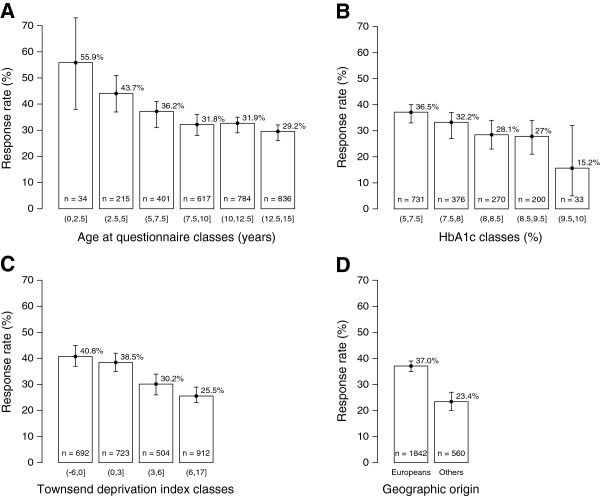
**Response rates according to: (A) age at the time of questionnaire completion (p =5 × 10** ^**−5**^ **); (B) HbA1c (p =0.006); (C) Townsend deprivation index (p =2 × 10** ^**−11**^ **); and (D) geographic origin (p =3 × 10** ^**−9**^ **).** Results are expressed as mean response rates for each class, T-bars represent the 95% confidence interval.

The second additive score simply added 4 scores arbitrarily defined according to age, HbA1c, TDI and geographic origin classes (Figure 1). For each of these variables, a score of “0” was arbitrarily assigned to participants in the classes with the lowest response rates; a score of “1” was assigned to participants in the classes with the second lowest response rate, etc. Minimum and maximum limits were 0 and 12 respectively for this additive score (mean =6.6).

**Table 1:** **Social characteristics of participants**

	Responders	Non-responders	p Value	
Characteristic	n =946	n =1886	Wilcoxon test	Logistic regression
Townsend deprivation index	2.85 ± 4.15 (2.00, −6.86-16.22)	4.04 ± 4.27 (3.87, −5.31-16.22)	1 × 10^−12^	2 × 10^−7^
Unemployment (% of those ≥16 yr and economically active)	10.30 ± 4.41 (9.39, 0.00-30.59)	11.49 ± 4.82 (10.90, 1.26-30.59)	4 × 10^−10^	0.005
Blue-collar workers (% of all households)	18.53 ± 7.69 (17.81, 0.00-50.00)	19.38 ± 7.80 (18.85, 0.00-50.00)	0.004	0.0002
White-collar workers (% of all households)	10.67 ± 7.52 (8.83, 0.00-50.09)	10.16 ± 7.07 (8.33, 0.00-38.10)	0.06	0.70
Non-car ownership (% of all households)	14.95 ± 11.33 (11.73, 0.00-74.81)	17.54 ± 11.47 (15.52, 0.00-69.42)	2 × 10^−11^	0.24
Farmers (% of all households)	1.69 ± 3.20 (0.42, 0.00-40.00)	1.38 ± 2.71 (0.18, 0.00-21.05)	2 × 10^−5^	0.85
Household overcrowding (% of all households)	1.50 ± 0.53 (1.43, 0.00-3.20)	1.65 ± 0.55 (1.59, 0.00-3.53)	6 × 10^−12^	0.02
Non-home ownership (% of all households)	36.67 ± 17.94 (32.99, 4.17-85.72)	41.22 ± 18.49 (40.14, 3.36-85.72)	7 × 10^−10^	0.24
Income (€/yr)	27784 ± 6677 (27298, 5902–67313)	26636 ± 6449 (25294, 5902–67509)	8 × 10^−6^	0.64
Access rate to high school graduate (% of all households)	37.88 ± 11.27 (36.73, 9.76-79.53)	37.19 ± 11.39 (35.31, 13.33-79.22)	0.04	0.10
Population density (inhabitants/km^2^)	3114 ± 5870 (1026, 7–49362)	3557 ± 5445 (1468, 2–49362)	5 × 10^−5^	0.45
Urban units index	3.92 ± 3.09 (4, 0–8)	4.36 ± 3.06 (6, 0–8)	0.0002	0.83
Europeans (% of all patients)	84%	73%	3 × 10^−9^	9 × 10^−5^

Values are means ± standard deviation (median, range).

All variables except origin were estimated on an environmental level (see Methods).

**Table 2:** **Personal and clinical characteristics of participants**

	Responders		Non-Responders		p Value	
Characteristic	n =946	n	n =1886	n	Wilcoxon/χ ^2^ test	Logistic regression
Sex (% males)	50%		53%		0.10	0.15
Age at T1D onset (yr)	6.28 ± 3.55 (5.90, 0.18-14.87)		6.17 ± 3.44 (5.78, 0.21-14.94)		0.55	0.62
Age at questionnaire (yr)	9.75 ± 3.44 (10.22, 1.31-14.99)		10.32 ± 3.18 (10.74, 0.42-14.98)		8 × 10^−5^	2 × 10^−5^
T1D duration at questionnaire (yr)	3.47 ± 3.07 (2.71, 0.00-14.49)		4.16 ± 3.16 (3.75, 0.02-13.70)		5 × 10^−9^	0.19
HbA1c (%)	7.57 ± 0.83 (7.5, 5.5-10.6)	526	7.81 ± 1.03 (7.7, 5.0-14.3)	1083	2 × 10^−5^	0.0005
Insulin dose (U/kg/d)	0.85 ± 0.27 (0.83, 0.11-1.89)	490	0.87 ± 0.28 (0.84, 0.14-3.73)	999	0.47	0.67
Body mass index (kg/m^2^)	17.14 ± 2.39 (16.64, 13.12-28.78)	494	17.35 ± 2.46 (16.82, 12.23-31.44)	998	0.053	0.36

Values are means ± standard deviation (median, range). Age at questionnaire was children’s age at the time the questionnaire was send.

*T1D*: type 1 diabetes, HbA1c: glycated hemoglobin.

### Statistics

Standard statistical methods were used to test the bivariate associations. A p <0.05 defined significance. Logistic-regression analyses were used to identify multivariate associations characterizing the patients whose parents returned the questionnaire, and a Cox proportional hazard model was used to study the time to questionnaire return. The variables entered into the multivariate regressions were those whose p values in the bivariate studies were <0.20.

## Results

Within 6, 12, 18 or 33 months, respectively, 867 (30.6%), 928 (32%), 938 (32.7%) and 946 (33.4%) questionnaires were returned.

Participants’ socioeconomic status based on their geocoded place of residence is reported in Table 1. Univariate analysis of available social variables showed that responders were overall more socially privileged than non-responders. Indeed, responders lived in places with lower population density, lower rates of unemployment, blue-collar workers, non-car ownership, household overcrowding and non-homeowners, and higher rates of white-collar workers, farmers and access to high school graduates. Their incomes were higher than those of non-responders. The TDI was higher for non-responders, with the lower the TDI, the better is the response rate: 36.6% for the more privileged vs 24.6% for the less privileged (Figure 1). Multivariate analysis (Table 1) retained TDI, unemployment rate, blue-collar workers, and household overcrowding as being significantly associated with returning questionnaire. The response rate was higher in participants of European than non-European origin.

The differences in personal and clinical characteristics of participants are reported in Table 2. T1D children of responders were younger than those of non-responders, and similar percentages of responders’ children were found in girls and boys (Table 2). Responders’ children had better HbA1c levels for equivalent daily insulin doses. Multiple regression analysis retained age and HbA1c as independent determinants of questionnaire response. Detailed analysis of response rates among the different classes of these 2 determinants (Figure 1) showed marked trends towards better response rates for lower age classes of T1D children and those with better glycemic control.

**Figure 2:**
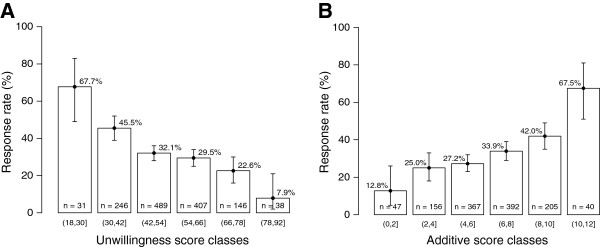
**Response rates according to: (A) Unwillingness score (p =1 × 10** ^**−10**^ **); (B) Additive score (p =3 × 10** ^**−9**^ **).** Results are expressed as mean response rates for each class, T-bars represent the 95% confidence interval.

**Figure 3:**
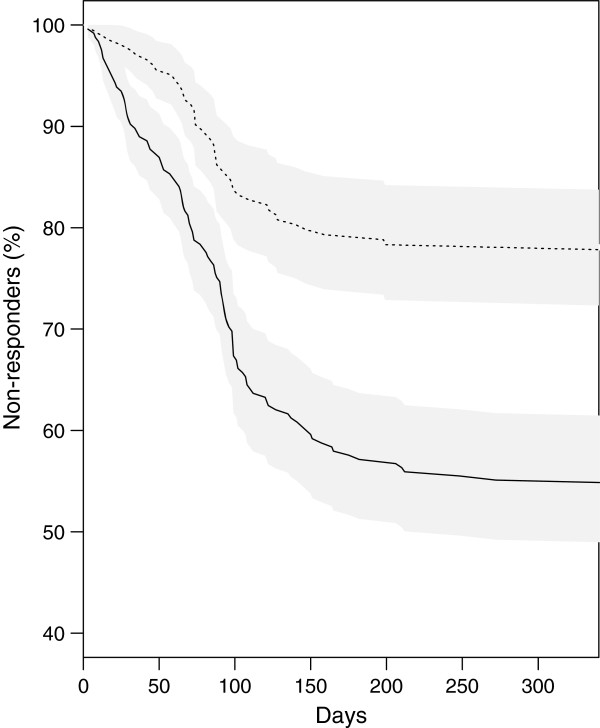
**Variation of the percentage of non-responders, as a function of time after receipt of the questionnaire.** Subjects with the best expected (8,12] (−) or lowest expected (0,4] (−−-) response rates are shown. Grey areas represent the 95% confidence intervals for each curve. The 2 curves differ significantly (log-rank test, p =0.002).

Finally, the 2 scores were highly predictive of response, and similar in terms of prediction (Figure 2). Because the additive score is simpler to use, we compared the variations of the response rates over time for the (8,12] and (0,4] additive score classes, respectively the “best expected response return” and the “lowest expected response rate” groups (Figure 3). The final difference was highly significant, with a 46% response rate for the former versus 22% for the latter (p =0.002).

Time to response varied widely, ranging from 1 day to 33 months, with 92% (n =867) of the responders returning the document within <6 months. This time was not associated with any of socioeconomic or personal variables considered (data not shown).

The completeness rates analyzed for responders was very high for filling out the entire questionnaire (mean computed for the 562 core questions: 96%) and each of its subparts (95% for part 1, 97% for part 2, 95% for part 3, and 96% for part 4). Notably, 79% (n =685) of the responders completed >95% of the questionnaire.

## Discussion

This study was undertaken to determine predictors of response or not to an environmental questionnaire by the parents of children with T1D. Analyses of returned documents revealed differences between responders and non-responders. The most important predictors of response (or not) were family’s socioeconomic environment, the child’s age at questionnaire completion and glycemic control, with responders’ children being more socially privileged, younger and having better HbA1c levels than those of non-responders.

Our observations that responders had higher socioeconomic status and education levels than non-responders are consistent with previous findings [24–29]. In the TEDDY study, whose population and subjects are comparable to ours, more educated mothers were more likely to return the questionnaire [30]. Employed people were also more readily participated in scientific studies [28, 29, 31].

In our study, European participants had a better response rate than patients of other origins. Ethnic status had previously been associated with responder’s education level and participation attrition [32, 33]. Like us, the TEDDY study also had to deal with issues of minority recruitment and retention in a pediatric cohort [34]. While some studies documented higher response rates among whites [28], a 2006 systematic review by Wendler et al. provided new evidence that ethnic minorities are as likely as majority groups to participate if invited to do so [35]. A possible explanation of our observation is inadequate presentation of the research project to these minority-group parents, leading to poor understanding of the challenges, perhaps added to the language barrier [36].

Children’s age at the time of the questionnaire reception was analyzed. It is interesting to note that parents of younger children were more likely to participate, perhaps reflecting greater concern about T1D or greater interest in understanding the causes of its early onset. Parents of older children had been living with T1D longer and might have developed some resilience to its presence and been less interested in its causes.

Our finding that responders’ children had better HbA1c levels for the same insulin dose seems consistent with the general trend in the literature about non-response, which more often shows a better health status in responders [24, 27, 37–41]. However, other study found better health status for non-responders [18]. In our case, parents who responded to the questionnaire could be considered more vigilant about their children’s health and, by extension, those who best managed T1D every day. For future analyses, it would be also interesting to measure the involvement of parents in the health care of their child, together with the parental perception of T1D susceptibility, severity, and perception of study participation benefits. Indeed, we lacked information on barriers which may avoid non-responders from responding to the questionnaire.

Not surprisingly, given the environmental questionnaire’s length which could be a major barrier [42], especially for families with lower education levels, only one-third of the families returned the completed environmental questionnaire. Although this small percentage may be considered worrying [43, 44], no relationship has been established between response rates and bias [45, 46]. It is quite possible that, for an inquiry generating a high response rate, the small percentage of non-responders could be critically different from that of the responders. A high response rate does not prevent a non-response bias. Hence, regardless of the response rate, all differences between responders and non-responders concerning a comprehensive set of demographic, clinical, behavioral pre-survey variables must be investigated. If no differences are found, one can probably deduce that the responders’ questionnaire results can be extrapolated to the whole population. However, when differences exist, extrapolation to the total population could be made possible by using appropriate statistical adjustment techniques for those variables. Even though the response rate is known to be lower when questionnaires are long [42], a subtle balance exists between a high response rate and little information, and a lower response rate but a comprehensive overview of the numerous candidate environmental factors possibly involved. We chose the latter, inasmuch we think that, with the additive score we developed, it should be possible in the future to increase response rates, by targeting those that we identified as being at high risk for non-response. Indeed, pre-contact (i.e., before sending the questionnaire), reminder postcards, monetary incentives or other strategies effectively improved response rates, as reviewed by Edwards et al. [47]. Nevertheless, the information provided by our scores could be important for two reasons: 1) the scores indicate how to recruit more easily new patients, and allow to allocate more work force to obtain questionnaires in patients living in « low probability of response » places, or with « low probability of response » clinical conditions; 2) these scores will be used in further analyses of a case–control comparisons, in a similar approach than those used with propensity scores in clinical research. A very simple estimation of such scores is possible, performing as well as the full regression equations, and could help investigators to improve their response rate.

## Conclusions

We think that the methodology of comparison of non-responders and responders described herein may contribute to successful implementation of comprehensive environmental epidemiological investigations of pediatric populations.

## Authors’ information

See the complete list of clinical centers participating in the Isis-Diab collaborative group (info@isis-diab.org) in Additional file 1: Table S1.

## Electronic supplementary material

Additional file 1: **List of the 99 diabetes centers participating to the Isis-Diab Network.** (DOCX 78 KB)

## Abbreviations

T1D: Type 1 diabetes
TDI: Townsend deprivation index
HbA1c: Glycated hemoglobin.
