# Supplementary material for: Comparison of a French pediatric type 1 diabetes cohort’s responders and non-responders to an environmental questionnaire
Source: BMC Public Health. 2014 Dec 3;14:1241. doi: 10.1186/1471-2458-14-1241 (PMC4326427; doi:10.1186/1471-2458-14-1241)
Supplement: Supplementary file 1 — Additional file 1: List of the 99 diabetes centers participating to the Isis-Diab Network. (DOCX 78 KB) [file 12889_2014_7479_MOESM1_ESM.docx]

**Comparison of a French pediatric type 1 diabetes cohort’s responders and non-responders to an environmental questionnaire**

**Additional files**

**Additional file 1.** List of the 99 diabetes centers by alphabetic order (according to city) participating to the Isis-Diab Network.

| Center (City (Department)) | Principal investigator |
| --- | --- |
| Aix-en-Provence (Pediatrics) | Dr Dominique Thevenieau |
| Alençon (Diabetology) | Dr Corinne Fourmy Chatel |
| Amiens (Diabetology) | Dr Rachel Desailloud |
| Amiens (Pediatrics) | Dr Hélène Bony-Trifunovic |
| Angers (Diabetology) | Dr Pierre-Henri Ducluzeau |
| Angers (Pediatrics) | Prof Régis Coutant |
| Armentières (Pediatrics) | Dr Sophie Caudrelier |
| Arras (Pediatrics) | Dr Armelle Pambou |
| Aurillac (Diabetology) | Dr Emmanuelle Dubosclard |
| Avignon (Pediatrics) | Dr Florence Joubert |
| Bar-le-Duc (Pediatrics / Diabetology) | Dr Philippe Jan |
| Belfort-Montbéliard (Pediatrics) | Dr Estelle Marcoux |
| Besançon (Pediatrics) | Dr Anne-Marie Bertrand/Dr Brigitte Mignot |
| Besançon (Diabetology) | Prof Alfred Penformis |
| Béthune (Pediatrics) | Dr Chantal Stuckens |
| Blois (Diabetology) | Dr Régis Piquemal |
| Bordeaux (Pediatrics) | Prof Pascal Barat |
| Bordeaux (Diabetology) | Prof Vincent Rigalleau |
| Boulogne-Billancourt (Pediatrics) | Dr Chantal Stheneur |
| Boulogne-sur-Mer (Pediatrics) | Dr Sylviane Fournier |
| Brest (Diabetology) | Prof Véronique Kerlan |
| Brest (Pediatrics) | Dr Chantal Metz |
| Brive (Pediatrics) | Dr Anne Fargeot-Espaliat |
| Caen (Diabetology) | Prof Yves Reznic |
| Cahors (Diabetology) | Dr Frédérique Olivier |
| Cambrai (Pediatrics) | Dr Iva Gueorguieva |
| Chartres (Diabetology) | Dr Arnaud Monier |
| Cholet (Pediatrics) | Dr Catherine Radet |
| Clamart (Pediatrics) | Dr Vincent Gajdos |
| Clermont-Ferrand (Pediatrics) | Dr Daniel Terral |
| Compiègne (Pediatrics) | Dr Christine Vervel |
| Contamine (Pediatrics) | Dr Djamel Bendifallah |
| Dijon (Pediatrics) | Dr Candace Ben Signor |
| Douai (Pediatrics) | Dr Daniel Dervaux |
| Draguignan (Pediatrics) | Dr Abdelkader Benmahammed |
| Dunkerque (Pediatrics) | Dr Guy-André Loeuille |
| Epinal (Pediatrics) | Dr Françoise Popelard |
| Gap (Diabetology) | Dr Agnès Guillou |
| Grenoble (Diabetology) | Prof Pierre-Yves Benhamou |
| Hyères (Pediatrics) | Dr Jamil Khoury |
| La Roche-sur-Yon (Pediatrics) | Dr Jean-Pierre Brossier |
| Laval (Pediatrics) | Dr Joachim Bassil |
| Le Creusot (Diabetology) | Dr Sylvaine Clavel |
| Le Havre (Pediatrics) | Dr Bernard Le Luyer |
| Le Kremlin-Bicêtre (Pediatrics) | Prof Pierre Bougnères |
| Le Mans (Pediatrics) | Dr Françoise Labay |
| Lens (Pediatrics) | Dr Isabelle Guemas |
| Lille (Pediatrics) | Prof Jacques Weill |
| Lille (Diabetology) | Dr Jean-Pierre Cappoen |
| Limoges (Diabetology) | Dr Sylvie Nadalon |
| Limoges (Pediatrics) | Dr Anne Lienhardt-Roussie |
| Lisieux (Diabetology) | Dr Anne Paoli |
| Lisieux (Pediatrics) | Dr Claudie Kerouedan |
| Lorient (Diabetology) | Dr Edwige Yollin |
| Lyon (Pediatrics) | Prof Marc Nicolino |
| Marseille (Pediatrics) | Prof Gilbert Simonin |
| Marseille Saint-Joseph (Diabetology) | Dr Jacques Cohen |
| Marseille CHU Nord (Diabetology) | Dr Catherine Atlan |
| Maubeuge (Pediatrics) | Dr Agnès Tamboura |
| Mérignac (Pediatrics) | Dr Hervé Dubourg |
| Mont-de-Marsan (Pediatrics) | Dr Marie-Laure Pignol |
| Montfermeil (Pediatrics) | Dr Philippe Talon |
| Nancy (Pediatrics) | Dr Stéphanie Jellimann |
| Nantes (Diabetology) | Dr Lucy Chaillous |
| Nantes (Pediatrics) | Dr Sabine Baron |
| Nice (Pediatrics) | Dr Marie-Noëlle Bortoluzzi |
| Nice Lenval (Pediatrics) | Dr Elisabeth Baechler |
| Nîmes (Pediatrics) | Dr Randa Salet |
| Niort (Pediatrics) | Dr Ariane Zelinsky-Gurung |
| Palavas Les Flots (Pediatrics) | Dr Fabienne Dallavale |
| Paris Hôtel-Dieu (Diabetology) | Dr Etienne Larger |
| Paris Lariboisière (Pediatrics) | Dr Marie Laloi-Michelin |
| Paris Saint-Louis (Diabetology) | Dr Jean-François Gautier |
| Pau (Pediatrics) | Dr Bénédicte Guérin/Dr Laure Oilleau |
| Pontoise (Pediatrics) | Dr Laetitia Pantalone |
| Reims (Diabetology) | Dr Céline Lukas |
| Rennes (Diabetology) | Dr Isabelle Guilhem |
| Rennes (Pediatrics) | Dr Marc De Kerdanet |
| Rouen (Pediatrics) | Dr Marie-Claire Wielickzo |
| Saint-Brieuc (Diabetology) | Dr Mélanie Priou-Guesdon |
| Saint-Etienne (Pediatrics) | Dr Odile Richard |
| Saint-Avold (Pediatrics) | Dr François Kurtz |
| Saint-Lo (Pediatrics) | Dr Norbert Laisney |
| Saint-Lo (Diabetology) | Dr Déborah Ancelle |
| Saint-Nazaire (Pediatrics) | Dr Guilhem Parlier |
| Saintes (Pediatrics) | Dr Catherine Boniface |
| Strasbourg (Diabetology) | Dr Dominique Paris Bockel |
| Tarbes (Pediatrics) | Dr Denis Dufillot |
| Toulon (Pediatrics) | Dr Berthe Razafimahefa |
| Toulouse (Diabetology) | Dr Pierre Gourdy |
| Toulouse (Pediatrics) | Dr Pierre Gourdy |
| Tours (Diabetology) | Prof Pierre Lecomte |
| Tours (Pediatrics) | Dr Myriam Pepin-Donat |
| Tremblay-en-France (Diabetology) | Dr Marie-Emmanuelle Combes-Moukhovsky |
| Troyes (Pediatrics) | Dr Brigitte Zymmermann |
| Valence (Pediatrics) | Dr Marina Raoulx |
| Valenciennes (Pediatrics) | Dr Anne Gourdin |
| Vienne (Pediatrics) | Dr Catherine Dumont |
| Villefranche-sur-Saône (Pediatrics) | Dr Michèle Chambon |
